# Role of Autophagy in *Haematococcus lacustris* Cell Growth under Salinity

**DOI:** 10.3390/plants11020197

**Published:** 2022-01-12

**Authors:** Daria A. Zharova, Alexandra N. Ivanova, Irina V. Drozdova, Alla I. Belyaeva, Olga N. Boldina, Olga V. Voitsekhovskaja, Elena V. Tyutereva

**Affiliations:** 1 Laboratory of Molecular and Ecological Physiology, Komarov Botanical Institute, Russian Academy of Sciences, ul. Professora Popova 2, 197376 St. Petersburg, Russia; odonato@bk.ru (D.A.Z.); ETutereva@binran.ru (E.V.T.); 2 Laboratory of Plant Anatomy, Komarov Botanical Institute, Russian Academy of Sciences, ul. Professora Popova 2, 197376 St. Petersburg, Russia; AIvanova@binran.ru; 3 Research Park, St. Petersburg State University, Universitetskaya emb., 7/9, 199034 St. Petersburg, Russia; 4 Laboratory of Ecology of Plant Communities, Komarov Botanical Institute, Russian Academy of Sciences, ul. Professora Popova 2, 197376 St. Petersburg, Russia; IDrozdova@binran.ru (I.V.D.); ABelyaeva@binran.ru (A.I.B.); 5 Laboratory of Algology, Komarov Botanical Institute, Russian Academy of Sciences, ul. Professora Popova 2, 197376 St. Petersburg, Russia; boldina@binran.ru; 6 Department of Artificial Intelligence Technology in Physiology and Medicine, Saint Petersburg Electrotechnical University “LETI”, ul. Professora Popova 5, 197022 St. Petersburg, Russia

**Keywords:** astaxanthin, autophagy, cell remodeling, *Haematococcus*, potassium, salinity

## Abstract

The microalga *Haematococcus lacustris* (formerly *H. pluvialis*) is able to accumulate high amounts of the carotenoid astaxanthin in the course of adaptation to stresses like salinity. Technologies aimed at production of natural astaxanthin for commercial purposes often involve salinity stress; however, after a switch to stressful conditions, *H.* *lacustris* experiences massive cell death which negatively influences astaxanthin yield. This study addressed the possibility to improve cell survival in *H.* *lacustris* subjected to salinity via manipulation of the levels of autophagy using AZD8055, a known inhibitor of TOR kinase previously shown to accelerate autophagy in several microalgae. Addition of NaCl in concentrations of 0.2% or 0.8% to the growth medium induced formation of autophagosomes in *H. lacustris*, while simultaneous addition of AZD8055 up to a final concentration of 0.2 µM further stimulated this process. AZD8055 significantly improved the yield of *H. lacustris* cells after 5 days of exposure to 0.2% NaCl. Strikingly, this occurred by acceleration of cell growth, and not by acceleration of aplanospore formation. The level of astaxanthin synthesis was not affected by AZD8055. However, cytological data suggested a role of autophagosomes, lysosomes and Golgi cisternae in cell remodeling during high salt stress.

## 1. Introduction

The xanthophyll astaxanthin has important applications as antioxidant in pharmacology as well as in fish and livestock farming. In contrast with natural astaxanthin, synthetic astaxanthin is not esterified and not recommended for nutraceutical purposes; therefore, the green microalga *Haematococcus lacustris* (formerly *H. pluvialis*) represents an attractive alternative for the commercial production of this carotenoid [1]. Under optimal conditions of autotrophic growth, astaxanthin is mainly located in the eyespot apparatus of the cells. However, during stress response *H. lacustris* cells synthesize high amounts of astaxanthin because it protects the alga from light and oxidation. Stress-induced astaxanthin accumulates in cytoplasmic lipid droplets (LDs) which are formed during astaxanthin synthesis in the course of transition of cells from their vegetative stage (either bi-flagellated macrozooids or aflagellate palmellae) via intermediate cells to stress-resistant aplanospores, also called haematocysts [2]. LDs derive from endoplasmic reticulum (ER); however, their stress-induced formation in *H. lacustris* cells occurs simultaneously with the degeneration of chloroplasts [2,3,4]. The biosynthetic precursor of astaxanthin, beta-carotene, is transported from chloroplasts to LDs via an as yet unknown mechanism. The biosynthesis of astaxanthin from beta-carotene requires several enzymatic reactions which are catalyzed by ketolase and hydroxylase. Astaxanthin resides in LDs in an esterified form. The astaxanthin-containing LDs shield nuclear DNA from oxidation and protect the whole cells from photooxidative damage, acting as a light screen (reviewed in [5]).

Aplanospores contain the highest amounts of astaxanthin as compared to other stages of the *H. lacustris* life cycle but they are resting cells and do not divide [2]. Therefore, in industry, in order to increase the yield of astaxanthin, the switch from favorable to stressful conditions is often preceded by a phase of extensive cell growth and biomass production. Mixotrophic cultivation [6], starvation of the culture [7] and salinity stress [8] are among the stresses used in astaxanthin production. While mixotrophic cultivation is effective but costly, application of NaCl to autotrophic cells is less expensive but causes massive cell death which diminishes the yield of astaxanthin (see, e.g., [9]).

In algae as well as in land plants, the retention of K^+^ in cells has been ascribed an important role in salinity tolerance [10,11,12]. Recently, induction of K^+^ loss from *Arabidopsis* root cells during salinity stress was shown to activate autophagy in these cells [13]. Stress-induced autophagy is a cytoprotective program enabling cell survival under stress conditions. In contrast, constitutive autophagy helps to remove damaged or unwanted cell constituents, for instance during the remodeling of cell structure. In algae, autophagy has multiple roles, and was shown to be induced in response to heavy metals, nitrogen starvation and salt stress [14,15,16]. In the microalga *Chlamydomonas reinhardtii*, autophagy is initiated concomitantly with the transformation of motile flagellate ‘macrozooid’ cells to round-shaped aflagellate ‘palmellae’ under stress conditions, suggesting a role of autophagy in cell remodeling [17].

In *C. reinhardtii*, a close relative of *H. lacustris*, autophagy was found to participate in lipid metabolism including triacylglyceride (TAG) synthesis [18,19,20,21]. Treatment of the cells with autophagy inhibitors such as concanamycin A (Conc A) interfered with the accumulation of LDs in N-starved *C. reinhardtii* but not in cells grown under standard conditions. Conc A does not inhibit the formation of autophagosomes, but inhibits proton-transporting ATPases, thus preventing the acidification of vacuole or lysosomes. As application of Conc A interfered with lipid synthesis in *C. reinhardtii,* Couso et al. (2017) suggested a role of vacuolar hydrolysis in LD formation [18]. A recent study has demonstrated a close interaction of autophagosomes with LDs in *C. reinhardtii* [22]. Furthermore, in mammalian cells, autophagy was found to participate in the biogenesis of LDs via TOR kinase and AMP-Kinase-mediated pathways [23]. The TOR kinase complex is a major activator of anabolic programs, growth and development, as well as an inhibitor of autophagy, in all eukaryotes [16]. In the microalgae *C. reinhardtii* and *Cyanidioschyzon merolae*, TOR signaling seems to play a critical role in the accumulation of TAG during stress [20,21]. Altogether, these results strongly suggest that also in *H. lacustris*, autophagy might be involved in the biogenesis of LDs. 

In the present study, we addressed the question whether an increase in the levels of autophagy in *H. lacustris* cells subjected to salinity stress could lead to better survival of the cells, thus potentially increasing astaxanthin yield. We also wanted to analyze whether an acceleration of autophagy would activate formation of LDs, and thereby increase the levels of astaxanthin synthesis. For this purpose, we added AZD8055 (AZD), an inhibitor of TOR kinase, to cell cultures simultaneously with NaCl, and monitored cell growth for the next five days. Previous studies where AZD had been applied to *C. reinhardtii* cells in concentrations between 0.7 µM and 2 µM had shown inhibition of cell growth and increased formation of LDs [21,24]. We used a three times lower concentration of AZD (0.2 µM) for two different levels of NaCl concentrations in the growth medium (0.2% or 0.8%, respectively; these levels had been suggested in an earlier study to represent a low and a high salinity stress, respectively, for *Haematococcus* [9]). Cell growth, formation of autophagosomes, as well as the levels of K^+^, Na^+^ and astaxanthin, were monitored in the cells during five days of cultivation. The results show that addition of 0.2 µM AZD to *H. lacustris* cells exposed to 0.2% NaCl enhanced cell yield (i.e., number of cells per volume culture unit) while in cells exposed to 0.8% NaCl, it rather exerted surplus stress instead of causing cytoprotection. In either case, the levels of astaxanthin synthesis per se were not affected by AZD. At the same time, cytological analyses suggested that in *H. lacustris* exposed to high salt stress autophagy participates in cell remodeling during formation of the shield consisting of astaxanthin-containing LDs.

## 2. Results

### 2.1. Treatment of Haematococcus lacustris Cells Exposed to 0.2% NaCl with AZD8055 Significantly Enhanced Cell Growth

**Figure 1:**
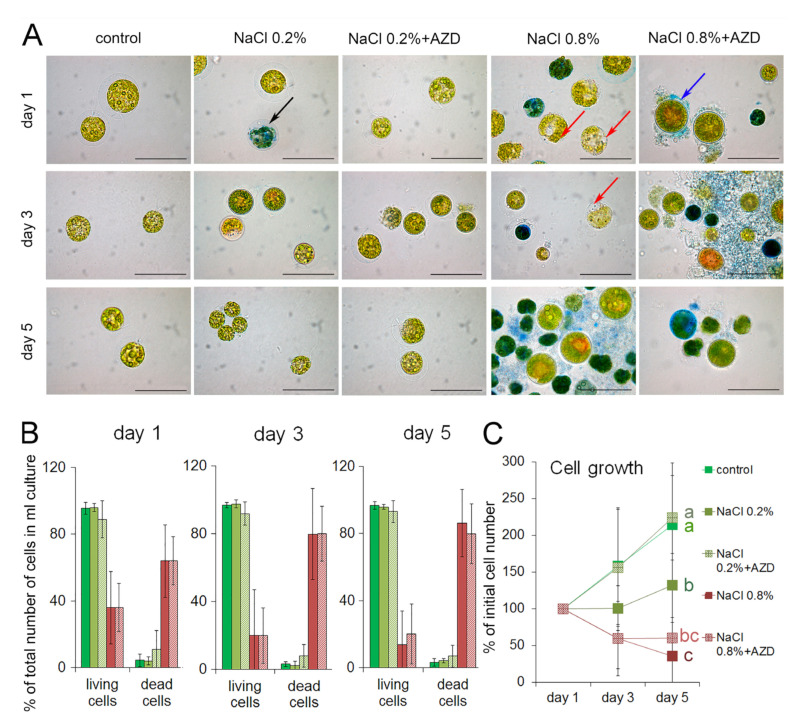
Growth of *Haematococcus lacustris* over five days in cultures containing 0.2% or 0.8% (*w*/*v*) NaCl, respectively, with or without addition of 0.2 µM AZD8055 (AZD). (**A**) Images of representative cells taken from the cultures at day 1, 3, or 5 of treatment as indicated and stained with Evans’ Blue to visualize dead cells. Black arrow points at a representative blue-stained cell considered ‘dead’. Red arrows point at extremely vacuolated cells. A blue arrow points at an intermediate cell. Bars denote 50 µm. (**B**) Changes in the proportions of living and dead cells as determined per unit of culture volume (mL); mean values from four independent experiments ± SD at day 1, 3, or 5 of treatment are shown. For the legend see C. (**C**) Growth dynamics at day 1, 3, or 5 of treatment; the cell density at day one was set to 100% for all cultures. Mean values from five independent experiments ± SD are given; different letters indicate significant differences between variants at the level of *p* < 0.05 according to one-way ANOVA and post hoc Tukey test.

*Haematococcus lacustris* cells were cultured in Optimized Haematococcus Medium (OHM; [25]) under low light (25 µmol m^−2^ s^−1^; see Materials and Methods for details) and continuous aeration. Transfer of logarithmically growing *H. lacustris* cells to growth medium containing 0.2% (*w*/*v*) NaCl did not cause any visible changes in cell morphology or growth after one day of treatment, and did not lead to an increase in cell death (Figure 1A,B). The same effect was observed when cells were transferred to medium containing 0.2% NaCl + AZD8055 (AZD; [26]). However, when cells were transferred to 0.8% (*w*/*v*) NaCl-containing medium, more than a half of the cells had died already after one day of treatment, as judged from the percentages of Evans’ Blue-stained cells in the total number of cells per ml culture, and the addition of AZD did not affect this death rate (Figure 1B).

After three or five days of treatment, the proportion of dead cells in cultures containing 0.2% NaCl in the growth medium did not increase as compared to control cells grown in the absence of NaCl regardless of the presence of AZD (Figure 1B). However, when the treatment proceeded, an effect of AZD on the growth rate could be observed by day 5. While the numbers of cells per culture volume unit in 0.2% NaCl-containing medium without AZD did not increase significantly during the course of the experiment, the addition of AZD restored the cell growth up to the levels of the control culture without salt (Figure 1C). In contrast, in media containing 0.8% NaCl, the proportion of dead cells continued to increase after the first day of treatment (Figure 1A,B). In these cultures, the cell numbers per culture volume unit decreased during the observation period, and this process was not affected by AZD (Figure 1C). In summary, while the addition of 0.2% NaCl interfered with growth during the observation period, it did not cause cell death, but the addition of 0.8% NaCl did.

In medium with 0.8% NaCl, i.e., in severely salt-stressed *H. lacustris* cultures, many cells showed the development of large vacuoles within one day of treatment (Figure 1A); this process was not affected by AZD. Cells with a similar morphology have been reported earlier by Yordanova and colleagues [27] for *C. reinhardtii* treated with the G-protein modulator mastoparan; those cells were suggested to undergo vacuolar programmed cell death (PCD) [27]. In salt-stressed *H. lacustris,* the appearance of these large vacuoles also seemed to precede cell death. The non-vacuolated *H. lacustris* cells in the cultures containing 0.8% NaCl accumulated astaxanthin already after one day of treatment, seemingly undergoing a transition to aplanospores; again, this process was not affected by AZD (shown in Figure 1A for 0.8% NaCl + AZD). No highly vacuolated cells could be detected in cells cultivated without added NaCl, or with 0.2% NaCl, after 24 h of treatment (data not shown). After three or five days of treatment, respectively, only intermediate cells and aplanospores were alive in cultures exposed to 0.8% NaCl, and the highly vacuolated cells had almost disappeared (data not shown).

### 2.2. Cellular K^+^/Na^+^ Ratio Decreased in Salt-Stressed H. lacustris with Increasing NaCl Concentrations

**Figure 2:**
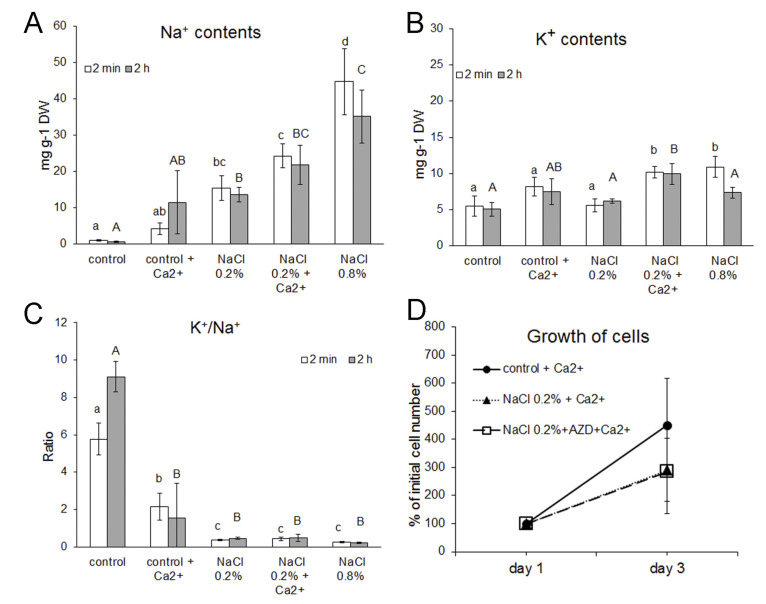
Levels of Na^+^ (**A**) and K^+^ (**B**) and K^+^/Na^+^ mass ratios (**C**) in *Haematococcus lacustris* cells after transfer to NaCl-containing medium. Mean values ± SD are shown. (**D**) Growth of *H. lacustris* in OHM containing 1 mM Ca^2+^ after transfer of the cells to fresh medium (control) or fresh medium containing 0.2% NaCl with or without addition of 0.2 µM AZD8055 (AZD). Data (mean values ± SD) from one experiment run in triplicates are shown; the cell density at day one was set to 100% for all cultures. Different letters indicate significant differences between variants at the level of *p* < 0.05 or lower according to one-way ANOVA and post hoc Tukey test; lowercase and uppercase letters are for measurements taken 2 min and 2 h after transfer, respectively.

To elucidate the dynamics of K^+^ and Na^+^ contents in *H. lacustris* cells grown under low (0.2% NaCl) or severe (0.8% NaCl) salt stress, the levels of these ions were determined 2 min and 2 h after transfer of the cells to NaCl-containing medium. While the levels of Na^+^ in control cells were negligible, they increased in the cells exposed to 0.2% NaCl, and the strongest accumulation of Na^+^ was observed in cells transferred to medium containing 0.8% NaCl (Figure 2A). K^+^ levels were approximately 5 mg g^−1^ dry weight (DW) in control cells grown in OHM (Figure 2B). In cells exposed to 0.2% NaCl, K^+^ contents were similar to those in control cells both after 2 min and after 2 h of exposure. However, when cells were transferred to medium containing 0.8% NaCl, K^+^ contents increased significantly within 2 min, but after 2 h they had decreased to the same levels as in cells in 0.2% NaCl-containing medium (Figure 2B). These data suggest that K^+^ uptake took place in the first phase of severe salt stress, probably as a mechanism to interfere with the extensive Na^+^ uptake (compare Figure 2B for control cells vs. cells exposed to 0.8% NaCl). Altogether, the cellular K^+^/Na^+^ ratio strongly decreased in cells subjected to both levels of salt stress as compared to control cells, and the strongest decrease was observed in severely stressed cells (Figure 2C).

Ca^2+^ ions have been suggested to protect cells from salt stress by improving K^+^ retention both in plants and algae [11,12]. Therefore, we compared K^+^ and Na^+^ contents in cells of *H. lacustris* grown in OHM containing 1 mM Ca^2+^ instead of 0.4 mM, as well as in cells exposed to low salt stress (0.2% NaCl) in medium containing 1 mM Ca^2+^. Prior to the experiments, *H. lacustris* was grown in OHM containing 1 mM Ca^2+^ for one week to allow adaptation of the cells to the new Ca^2+^ concentration. Increase of Ca^2+^ levels in standard growth medium did not affect K^+^ or Na^+^ levels in control cells; however, the levels of K^+^ were significantly enhanced in cells exposed to 0.2% NaCl in presence of 1 mM Ca^2+^. Altogether, additional Ca^2+^ in the growth medium led to a decrease of the K^+^/Na^+^ ratio in control cells but did not influence this ratio in cells exposed to 0.2% NaCl (Figure 2C). In one experiment, cell growth in OHM medium containing 1 mM Ca^2+^ was followed for three days (Figure 2D), and, strikingly, stimulation of growth was observed in all cultures (compare Figure 1C and Figure 2D), while the stimulatory effect of AZD on cell growth seemed to disappear. Addition of AZD to cells grown in the presence of 0.2% NaCl did not affect K^+^ or Na^+^ levels (data not shown).

### 2.3. OJIP-Kinetics in H. lacustris Cells Were Influenced by 0.8% NaCl in Growth Medium 

**Figure 3:**
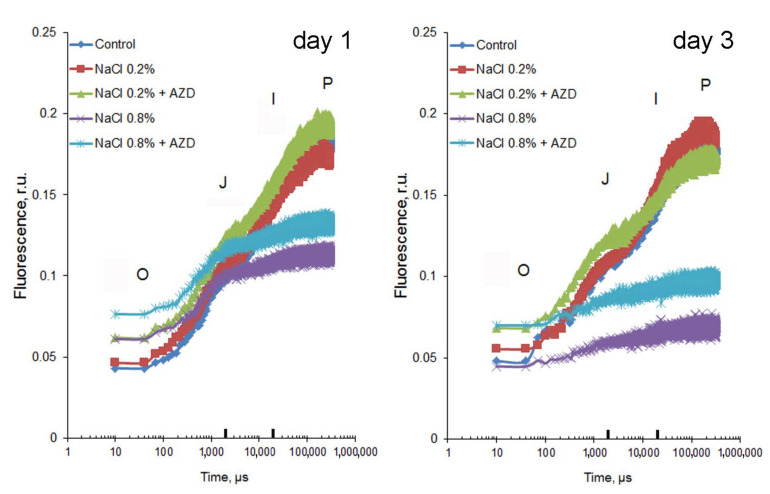
Effects of salinity and/or addition of AZD8055 (0.2 µM) on the fluorescence induction parameters (OJIP curves) in *Haematococcus lacustris* cells exposed for 1 or 3 days to different levels of NaCl (0.2% or 0.8%, respectively) either in the presence or absence of AZD8055 (AZD). Each curve represents the average of 10–24 replicates. Black marks on the X-axis correspond to 2 ms (‘J’-step) and 20 ms (‘I’-step), respectively.

As shown above, addition of 0.2% NaCl to *H. lacustris* cells led to retardation of cell growth (Figure 1C) and accumulation of Na^+^ in the cells (Figure 2A). However, the Na^+^ levels of these cells were similar to those of cells grown in the absence of Na^+^ but in the presence of 1 mM Ca^2+^ (Figure 2A) suggesting that such levels might represent an adaptive response and not a stress response per se. The analysis of fluorescent transients is a highly informative method for estimating the influence of stress factors on photosynthesis [28]. Chlorophyll a fluorescence induction curves (OJIP-kinetics) after 1 or 3 days of cell exposure to various levels of NaCl and AZD were similar in control cells and in cells exposed to 0.2% NaCl with and without addition of AZD (Figure 3); at the same time, exposure to 0.8% NaCl strongly influenced the OJIP-curves both in the absence and presence of AZD (Figure 3). 

Analyses of changes in several JIP-test parameters (Table 1) further suggested that *Haematococcus lacustris* cells experienced stress upon addition of 0.8%, but not 0.2%, NaCl to the growth medium. Cells responded to 0.8% NaCl with an increase in F_O_, a decrease in F_M_, a decrease in the maximal quantum yield φ_O,_ an increase in T_FM_ indicating a partial block of electron transfer from PSII to PQ, a drop in ψ_O_ indicating reduced donation of electrons from PSII to the PQ-pool, a decrease in S_M_ indicating a drop in PQ pool size (Table 1). Notably, addition of AZD on top of NaCl (both at 0.2% and 0.8% levels) rather enhanced, than alleviated, stress exerted by salinity on photosynthesis (Table 1).

### 2.4. Addition of AZD Increased Formation of Autophagosomes in Salt-Stressed H. lacustris Cells

As shown above, exposure of cell cultures to 0.2% NaCl inhibited cell growth rate while addition of 0.2 µM AZD to such cells restored it up to the levels found in control cells (Figure 1C). This effect was not due to improved cell survival as AZD did not influence the percentages of dead vs. living cells (Figure 1B). Since AZD is an inhibitor of TOR kinase, and both TOR inhibition [16] as well as high level of salt stress [29] was shown to inhibit growth and stimulate autophagy in algae, we asked the question whether the low AZD concentration used in our experiments was sufficient to enhance autophagy at low levels of salt stress. 

**Figure 4:**
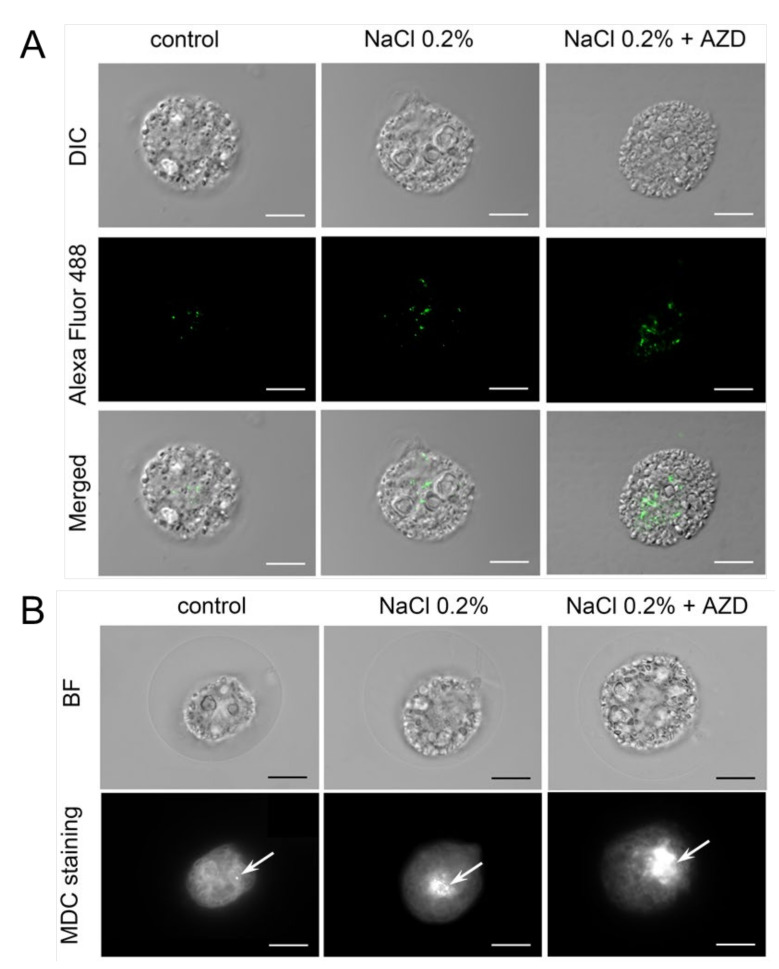
Visualization of autophagosomes in cells of *Haematococcus lacustris* cultured for three days either in OHM (control) or in OHM supplemented with 0.2% NaCl with or without addition of 0.2 µM AZD8055 (AZD), respectively. (**A**) Immunofluorescence with anti-CrATG8 antibody. (**B**) Staining with monodancyl cadaverine (MDC) after incubation of the cells with 1 µM concanamycin A for 24 h. DIC, Differential Interference Contrast; BF, bright field. White arrows point at a single autophagosome (control) or at an aggregation of autophagosomes. Bars denote 10 µm.

To this aim, autophagosomes were visualized in cells after three days of treatment using immunofluorescence (Figure 4A). While autophagosomes could be detected in non-stressed control cells, more autophagosomes were seen in salt-stressed cells, and addition of AZD seemed to further increase their numbers (Figure 4A). To prove this, we used an inhibitor of V-ATPases, Conc A, which does not interfere with autophagosome formation but prevents their fusion with the lytic compartments, thus allowing accumulation of autophagosomes in the cytosol. After incubation of the cells with Conc A for 24 h, autophagosomes were visualized by staining with monodancyl cadaverine (MDC) [30]. Multiple autophagosomes was detected in NaCl-treated cells around the nucleus but not in control cells, and even more autophagosomes were seen in cells where AZD was supplied together with NaCl (Figure 4).

### 2.5. The Levels of Astaxanthin in Salt-Stressed H. lacustris Cells Were Not Affected by AZD8055 

**Figure 5:**
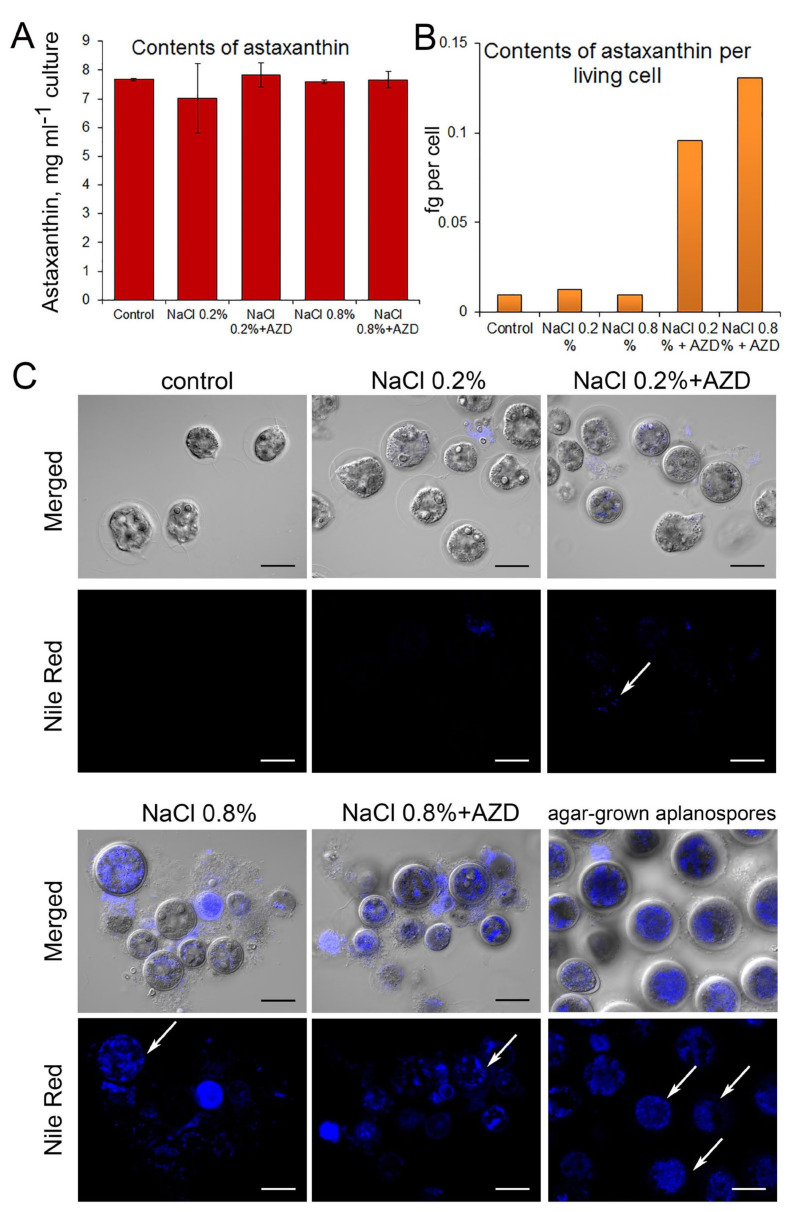
Levels of astaxanthin in *Haematococcus lacustris* cultures (**A**,**B**), and visualization of lipid droplets using Nile Red dye (**C**) after five days of treatment. One representative experiment is shown (B,C). Cells were cultured for five days either in OHM (control) or in OHM supplemented with NaCl (0.2% or 0.8%) with or without addition of 0.2 µM AZD8055 (AZD), respectively. Astaxanthin-containing red-colored aplanospores were collected from cultures grown on 1.5% OHM agar 2 months after streaking out liquid cultures on plates and used as a positive control of Nile Red staining. White arrows point at stained lipid droplets. ‘Merged’ denotes superposition of Nile Red signals (lower row) with images of the same cells taken using Differential Interference Contrast (DIC). Bars denote 20 µm.

Analyses of the levels of astaxanthin were performed at the fifth day of treatment; however, no visible changes in the color of cultures could be detected at this time point and, accordingly, the levels of astaxanthin per culture volume unit were essentially similar (and very low) in all treatments (Figure 5A). If calculated per living cell, by far the highest levels of astaxanthin were found in cultures exposed to 0.8% NaCl (Figure 5B). To answer the question whether AZD could affect the rate of LD formation, we analyzed cells at the third day of treatment; red aplanospores grown on agar plates were taken as a positive control as these cells were rich in astaxanthin-containing LDs. Staining of the cells with the lipid-specific dye Nile Red showed that LDs were almost absent from the cells exposed to 0.2% NaCl while exposure to 0.8% NaCl led to the appearance of multiple LDs in the absence as well as the presence of AZD (Figure 5C). 

### 2.6. Autophagosomes and Autolysosomes Were Abundant in H. lacustris Cells Subjected to High Salt Stress

**Figure 6:**
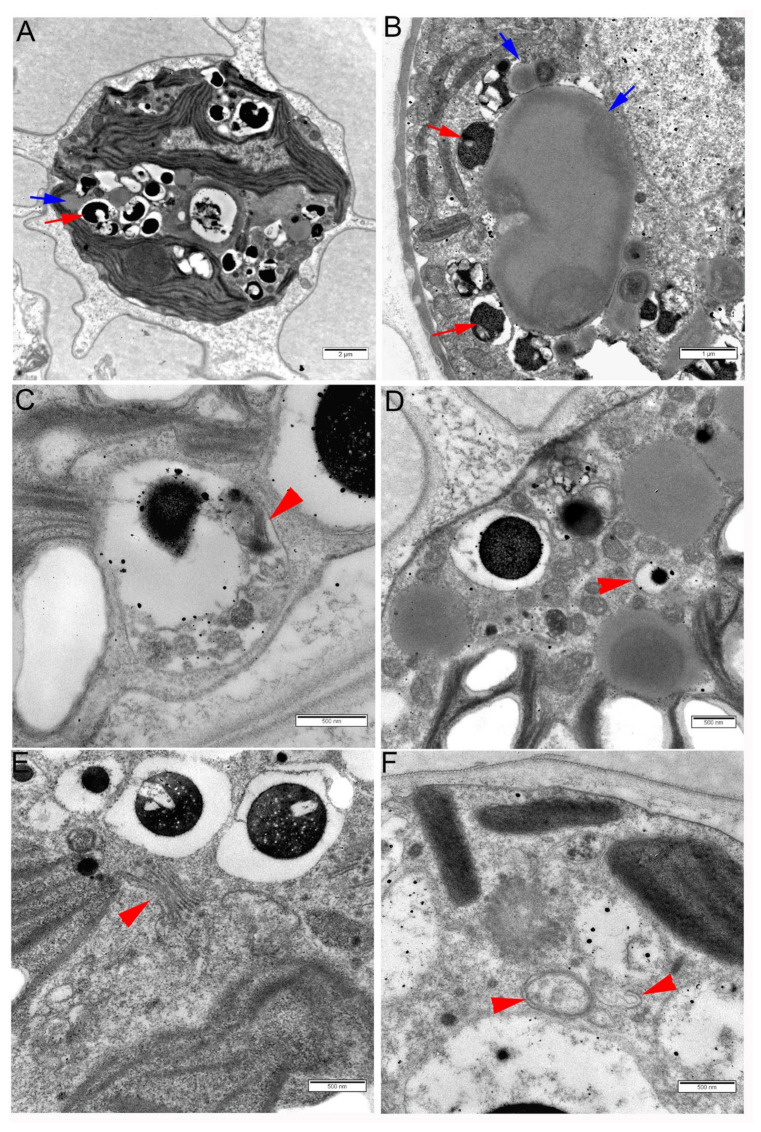
Ultrastructure of cells of *Haematococcus lacustris* cultures after three days of exposure to 0.8% NaCl. (**A**) A whole cell; red arrow points at an osmiophilic granule probably containing carotenoids including astaxanthin; blue arrow points at a lipid droplet. (**B**) Part of a cell showing lipid droplets (blue arrows) with ‘carotenoid bodies’ docking on them (red arrows). Osmiophilic ‘carotenoid bodies’ are located within single-membrane small vacuoles probably representing autolysosomes. (**C**) An autolysosome containing thylakoid remnants (red arrowhead). (**D**) Part of a cell showing electron-translucent lipid droplets, an electron-dense osmiophilic ‘carotenoid body’ within an autolysosome, and a small ‘carotenoid body’ surrounded by a double-membrane autophagosome (red arrowhead). (**E**) Several ‘carotenoid bodies’ in the vicinity of Golgi cisternae (red arrowhead). (**F**) Autophagosome (left red arrowhead) and a structure probably representing an omegasome with an expanding phagophore (right red arrowhead).

To further analyze changes induced in the cell organelles of *H. lacustris* by exposure to high levels of NaCl, transmission electron microscopy (TEM) was applied. Altogether, addition of AZD did not cause any change in cell structure (data not shown). In cells exposed to 0.8% NaCl for three days, many electron-translucent LD were visible in TEM, as well as multiple electron-dense bodies (Figure 6A,B). As OsO_4_ binds readily to double bonds of unsaturated lipids but very poorly to saturated lipids, we interpreted the observed LDs as organelles rich in TAG and the electron-dense bodies as rich in carotenoids, probably astaxanthin or its precursors, similar to the interpretations made by Wayama et al. and He et al. [2,3]. Strikingly, these ‘carotenoid bodies’ mostly occurred inside small vacuoles (Figure 6C,D) which was in contrast to the observations of Wayama et al. [2] but consistent with the ‘astaxanthin granules’ described by He et al. [3]. The smaller ones of these ‘carotenoid bodies’ were surrounded by double membranes suggesting that they were located in autophagosomes; larger ’carotenoid bodies’ were surrounded by a single membrane and probably represented autolysosomes (Figure 6C–E). In some of these bodies, remnants of thylakoids could be observed (Figure 6C). Multiple Golgi cisternae were present in the cells, and formation of autophagosomes was observed in their close vicinity; expanding phagophores could be detected (Figure 6E,F). 

## 3. Discussion

AZD8055 (AZD) is a highly selective inhibitor of TOR kinase; its mechanism of inhibition is not allosteric like that of rapamycin but it competes with ATP binding. In the present study, AZD was applied to salt-stressed *Haematococcus lacustris* cells, and its effects on cell growth, autophagy levels, as well as on the accumulation of astaxanthin were analyzed. Growth of *H. lacustris* was monitored for five days (i.e., 120 h) after a single addition of AZD to the growth medium. We assume that AZD remained stable in the medium over most of the observation period as it can be administered orally in mammals; AZD has been applied to mammalian cells for as long as 96 h [26]. 

Previous studies on cultured microalgae have monitored the effects of TOR inhibition by application of rapamycin, AZD or torin over a period of 24 h to 48 h. They showed that already after 24 h, the treatment led to growth arrest in the green alga *Chlamydomonas reinhardtii* [14,19,20,21,24] as well as in the red alga *Cyanidioschyzon merolae* [20]. Other common responses elicited by TOR inhibitors in non-stressed as well as stressed cells of *C. reinhardtii* were accumulation of neutral lipids and induction of autophagy [14,19,20,21,24]. However, in contrast to the studies cited above, AZD caused acceleration, rather than inhibition, of cell growth in *H. lacustris* exposed to low salt stress (0.2% NaCl in the growth medium), and had no effect on the growth of severely salt-stressed cells (0.8% NaCl) (Figure 1C). As AZD did not lead to a decrease in cell death (Figure 1B), the restored growth of cells exposed to low salt stress in the presence of AZD can be attributed to an increase in cell divisions rather than in cell survival. In contrast, for non-stressed cells of *C. reinhardtii*, addition of AZD seemed to inhibit cell divisions [24]. This discrepancy might be due to the difference in AZD concentrations applied in both cases: 0.7 µM were used by Ford and collaborators [24] while 0.2 µM were used in the present study. Interestingly, overexpression of a papain-like cysteine protease from *H. lacustris* in *C. reinhardtii* caused an abnormal increase in growth rate of transformants, suggesting that dysregulation of catabolic processes might influence the cell cycle [31].

Although formation of LDs and increase in TAG were observed in *C. reinhardtii* already 24–48 h after application of 0.7–2 µM AZD or 1 µM rapamycin [19,20,21,25], we did not observe accumulation of LDs after three days of exposure of salt-stressed *H. lacustris* cells to 0.2 µM AZD over the levels found without addition of AZD (Figure 5). However, we did observe an increase in autophagic flux in *H. lacustris* cells exposed to low levels of salt stress upon addition of 0.2 µM AZD (Figure 4). Thus, while the low concentration of AZD used in the present study was sufficient for the activation of autophagy, the concentration seems to have been too low to trigger an increase in neutral lipids and the formation of LDs in the same cells even after three days of exposure. In green algae, core components of the TOR kinase complex are encoded by single copy genes [32,33]; therefore, the contrasting effects of AZD on growth of *H. lacustris* compared to other microalgae cannot be related to the activation of alternative TOR isoforms, but have to depend on the concentration of the inhibitor. However, it should be taken into account that salt tolerance can differ between individual strains of *H. lacustris* [34] and between different microalgae, which might have affected the results.

Nevertheless, the stimulatory effect of low AZD concentrations on the growth of cells exposed to low salt stress is striking. In contrast to our original hypothesis, it could not be related to improved cell survival due to activation of autophagy leading to increased transition of vegetative cells into stress-resistant aplanospores. The fact that the stimulatory effect disappeared in the presence of enhanced Ca^2+^ levels (Figure 2D) suggests that signaling events might be involved. Although high Ca^2+^ levels in the growth medium did not lead to an increase in the cellular K^+^/Na^+^ ratio in cells of *H. lacustris* exposed to low levels of salt stress (Figure 2C), they caused an increase in the growth rate of non-stressed cells as well as of cells subjected to 0.2% NaCl. This is consistent with an earlier observation of a positive effect of Ca^2+^ on cell growth and astaxanthin accumulation for salt-stressed (0.25% NaCl) *H. lacustris* [8]. Similarly, in the microalga *Monoraphidium* sp., cytosolic levels of GABA and Ca^2+^ have been suggested to represent key players in the enhancement of cell growth, stress resistance and lipid accumulation under salt stress via affecting ROS signaling [35].

Recent studies have placed TOR in the center of redox signaling in microalgae: in *Chlamydomonas* cells, cysteine and methionine pools were affected by rapamycin [36], and a network of cysteine-containing proteins could be shown to undergo oxidation during the inhibition of TOR activity [25]. In combination with the results observed, this opens the question whether the inhibition of TOR by low concentrations of AZD might act upstream of Ca^2+^ sensing and thus be able to enhance growth under salt stress. Another explanation for the results of this study might be that the increase of autophagy caused by low AZD concentrations improves cell homeostasis due to better scavenging of damaged cell components [37], and thus promotes cell divisions. Further studies of the interactions between TOR signaling, autophagy, and GABA- and Ca^2+^-mediated signaling in *H. lacustris* are necessary to find out whether these signaling networks can be manipulated to promote astaxanthin synthesis [38].

It should be noted that we did not detect an increase in astaxanthin production per culture volume unit in salt-stressed cells either in the presence or in the absence of AZD during the five days of observation. This is in agreement with other studies where accumulation of astaxanthin began after five or more days of salt stress [8,9]. However, in cells exposed to high salt stress, dramatical remodeling of the ultrastructure related to the synthesis of astaxantin was observed (Figure 6). Such remodeling has been described before [2,3] and is characterized by a dismantling of chloroplasts, accumulation of lipid bodies and appearance of osmiophilic astaxanthin granules which at the later stages fuse with lipid bodies forming ‘astaxanthin oil droplets’ [2]. However, while the biochemistry of astaxanthin synthesis has been characterized [5], compartmentation of its stages between cell organelles is far from understood. We observed that in many cases the prevailing location of osmiophilic bodies was in single-membrane organelles which resembled autolysosomes; smaller osmiophilic granules were often located in autophagosomes surrounded by double membrane. It should be noted that the single large lytic vacuole typical for plant cells is absent from *H. lacustris* as well as *C. reinhardtii* cells: only small contractile vacuoles are present [2,39] and lytic compartments are represented by lysosomes and lysosome-derived organelles such as acidocalcisomes [40]. Thus, it is probable that macroautophagy in cells of these algae has structural similarity to animal cells in that the autophagic flux ends in lysosomes and not in the lytic vacuole. Furthermore, we often observed formation of autophagosomes in the close vicinity of Golgi stacks suggesting that these organelles were closely related in *H. lacustris* (Figure 6). Recently, a role of Golgi network in delivering membranes to autophagosomes has been revealed for mammalian cells [41] suggesting that this is another ‘animal’ trait of autophagosome biogenesis in algae. 

Although the data presented in this study strongly suggest that in *H. lacustris,* autophagy might be required for astaxanthin synthesis (Figure 6), the details of its role are still unclear. The source of the large ‘carotenoid bodies’ that might represent advanced stages of the degradation of parts of chloroplasts (Figure 6C) awaits further confirmation. Close association of autophagosomes with LDs had already been shown for *C. reinhardtii* [22]. The docking of ‘carotenoid bodies’ surrounded by autophagosomal or autolysosomal membranes at LDs might represent the delivery of astaxathin, or its precursors, to LDs, in order to form astaxanthin-containing oil droplets. In this case, these organelles might contain some enzymes of astaxanthin synthesis. It is tempting to speculate that autophagosomes might provide the link between the dismantling of chloroplasts, synthesis of astaxanthin from beta-carotene and delivery of astaxanthin to LDs. However, at this point, it is still possible that the observed autolysosome-like structures are related to salt stress-induced degradation of organells [29] and not to astaxanthin synthesis. Further studies are required to elucidate the compartmentaliztion of the different stages of astaxanthin and lipid syntheses in cells of *H. lacustris*.

## 4. Conclusions

This study has shown that low concentrations of AZD8055 can increase the growth of *H. lacustris* cultures under condition of low, but not severe, salt stress, which reveals a new aspect of the role of TOR kinase signaling in stress resistance of this microalga. Furthermore, this study reveals a role of autophagy-related structures in the process of cell remodeling during astaxanthin synthesis in cells of *H. lacustris* subjected to severe salt stress. 

## 5. Materials and Methods

### 5.1. Plant Material and Growth Conditions

*Haematococcus lacustris* Flotow strain IMBR-1 was isolated from subtropical valleys of the Black Sea region [7]. The cells were cultured in 0.3 L Erlenmeyer flasks in 200 mL of modified OHM (Optimized Haematococcus Medium; [25]) which contained KNO_3_ (410 mg L^−1^), Na_2_HPO_4_ (30 mg L^−1^), MgSO_4_ ∙ 7H_2_O (246.5 mg L^−1^), CaCl_2_ (48.1 mg L^−1^), FeC_6_H_5_O_7_ ∙ 5H_2_O(2.62 mg L^−1^), CoCl_2_ ∙ 6H_2_O (0.011 mg L^−1^), CuSO_4_ ∙ 5H_2_O (1.012 mg L^−1^), Cr_2_O_3_ (0.076 mg L^−1^), MnCl_2_ ∙ 4H_2_O (0.989 mg L^−1^), Na_2_MoO_4_ ∙ 2H_2_O (0.12 mg L^−1^), Na_2_SeO_2_ (0.008 mg L^−1^), ZnSO_4_ ∙ 7H_2_O (0.1 mg L^−1^) and thiamine (17.5 mg L^−1^). In one series of experiments, the concentration of CaCl_2_ in the medium was increased in that 111.08 mg L^−1^ were added instead of 48.1 mg L^−1^. 

Cells were grown at 24 °C and constantly aerated using a compressor (Schego, SCHEGO Schemel & Goetz GmbH & Co KG, Offenbach, Germany). White light was provided by Osram 36W fluorescent tubes with a PPFD (400–700 nm) of 25 µmol m^−2^ s^−1^ including a PFD-B (400–500 nm) of 5 µmol m^−2^ s^−1^, a PFD-G (500–600 nm) of 13 µmol m^−2^ s^−1^, and a PFD-R (600–700 nm) of 7 µmol m^−2^ s^−1^, respectively. In the region of 700–780 nm, a negligibly low PFD-FR of 0.8 µmol m^−2^ s^−1^ was measured using a PG100N handheld spectra PAR meter (UPRtek, Miaoli County, Taiwan). After two weeks of growth, cells from the culture volume of 50 mL were transferred to 200 mL of fresh OHM by means of centrifugation and resuspension in fresh medium and incubation continued as described above. The cell density at the end of a two-week cultivation period reached 200,000–500,000 cells per ml culture.

### 5.2. Induction of Salinity Stress and Treatment with Inhibitors 

After seven days of growth (in the middle of logarithmic phase), cells were centrifuged at 600× *g* for 5 min and resuspended in OHM without additives (control), or in OHM containing NaCl at a final concentration of 0.2% (*w*/*v*) or 0.8% (*w*/*v*), respectively, or in OHM containing 0.2% or 0.8% of NaCl supplemented with AZD8055 ((5-(2,4-bis((S)-3-methylmorpholino)pyrido[2,3-d]pyrimidin-7-yl)-2-methoxyphenyl)methanol; MedChemExpress) at a final concentration of 0.2 µM. Cell density at the beginning of experiments was 50,000–150,000 cell per ml culture depending on the experiment. The cultures were then grown as described above. Cells were collected for analyses after 1, 3, and 5 days of cultivation, respectively.

### 5.3. Microscopic Studies

Cells were observed and photographed using an Olympus BX51 microscope (Olympus Deutschland GmbH, Hamburg, Germany) equipped with a ColorView II digital camera and Cell ^F image analytical software (Olympus Soft Imaging Solutions, Münster, Germany). Cell growth was estimated using a cell counting chamber and a 10× objective.

### 5.4. Determination of Cell Viability

One ml of culture was supplemented with 100 µL of Evans’ Blue (0.5% (*w*/*v*) in water), incubated for 15 min, washed three times with fresh medium and observed under the microscope. Blue-stained cells were considered dead; determination of percentages of dead and living cells in the culture was performed using a cell counting chamber.

### 5.5. Determination of Cellular K^+^ and Na^+^ Levels

Cells (corresponding to 100 mL of the culture after 12 d growth for each sample) were collected by centrifugation and resuspended in a similar volume of OHM (control) or in OHM supplemented with 0.2% or 0.8% NaCl, respectively. After 2 min or 2 h depending on the experiment, the cells were centrifuged again, the pellets were weighted and immediately placed in a thermostat at 110 °C for 1 h. Then the temperature was set to 65 °C, and drying continued until the dry weight reached constant values. Dried cells were then transferred to acid-washed quartz beakers which were placed first in a muffle furnace at 450 °C and then in a desiccator for cooling. The ash was dissolved in a mixture of 1.5 M HNO_3_ and 3.71 M HCl. The concentrations of K^+^ and Na^+^ were determined using atomic absorption spectrometer Quantum-AFA (Cortec, Moscow, Russia). Performance of the instrument was checked by analyzing the reference standard material solutions: State Standard Samples GSO 8092–94 and GSO 8062–94. For each variant, five cell samples from five different cultivation flasks were analyzed.

### 5.6. Chlorophyll (Chl) a Fluorescent Transients

Fluorescence measurements were performed using a DUAL-PAM 100 and a DUAL-K25 quartz glass cuvette (Walz, Germany). Three aliquots from each culture of *H. lacustris* were transferred to Eppendorf tubes, and the cell density was assessed by cell counting and equalized to about 10.000 cells per 1 mL culture. The aliquots were sequentially transferred to the DUAL-K25 cuvette and dark-adapted for 10 min prior to measurements. OJIP measurements were performed using saturating red light of 3000 µE m^−2^ s^−1^ intensity and 300-ms polyphasic fluorescence trigger mode. Fluorescence transients were recorded with a data acquisition rate of 1 per 10 μs. The fluorescence signal at 40 μs after the onset of illumination was considered as F_O_. Recorded DUAL-PAM 100 data in the CSV format were analysed using pyPhotoSyn software [42].

### 5.7. Immunofluorescence

The protocol of [43] was used with modifications. Cells (4 mL culture) were collected by centrifugation and resuspended in 300 µL of 4% (*w*/*v*) paraformaldehyde (PFA, Sigma -Aldrich, Chemie GmbH, Steinheim, Germany) in Phosphate-Buffered Saline (PBS) buffer containing (final concentrations) 150 mM NaCl, 3 mM KCl, 11 mM Na_2_HPO_4_ and 2 mM KH_2_PO_4_ at pH 7.4. Cells were fixed overnight at 4 °C. All further changes of solutions were performed by means of sedimentation via short centrifugation and removal of supernatant followed with gentle resuspension of the cell pellet in the corresponding solution. All incubations were performed on a mini-rocker MR-1 shaker (Biosan, Riga, Latvia). The cells were washed twice with PBS, then incubated with 300 U mL^−1^ DNase-free proteinase K (Promega, Madison, WI, USA) for 30 min at 37 °C, then washed three times with PBS. Then, cells were incubated in 100% methanol precooled to −20 °C for 20 min and then washed twice with PBS. This was followed by incubation in blocking buffer (5% (*w*/*v*) globulin-free bovine serum albumin in PBS) for 1 h. Cells were washed twice with PBS and transferred to 500 µL of PBS-T solution (0.1% (*v*/*v*) Tween-20 in PBS buffer) supplemented with 1 µL of primary antibody AS14 2769 (Agrisera, Vännäs, Sweden) developed in rabbit against *C. reinhardtii* ATG8 protein. Cells were incubated with the primary antibody for 1 h at room temperature, followed by six washes with PBS-T. Then, cells were incubated with goat anti-rabbit secondary antibody conjugated with Alexa Fluor 488 (Invitrogen) for 1 h and washed six times with PBS. Cells were then mounted on slides with Prolong Gold Antifade reagent and incubated for 24 h in darkness. Cells were imaged using confocal laser scanning microscope LSM 780 and Zen 2.1 software (Carl Zeiss, Oberkochen, Germany). Alexa Fluor 488 was excited with an argon multilinear laser at 3% of the maximal intensity of 35 mV, and the signal was detected in the range of 510–540 nm at optical section thickness of 10 µm.

### 5.8. Visualization of Autophagosomes Using MCD and of Lipid Droplets Using Nile Red

On day 3 of experiments, 4 mL of culture were taken from each flask. Concanamycin A (Conc A; Sigma-Aldrich, St.-Louis, MO, USA) was prepared as a 100 µM stock solution in dimethylsulfoxide (DMSO) and added to the cells up to final concentration of 1 µM. The cells were incubated for 24 h with gentle shaking. The cells were then fixed in 4% PFA in 0.1 M potassium phosphate butter (PB) at pH 7.4 for 15 min and washed with PB. Then, the cells were incubated with 0.1% Triton-X-100 in PB for 15 min and washed with PB. Monodancyl cadaverine (MDC; Sigma-Aldrich, St.-Louis, MO, USA) was prepared as stock solution of 5 mM in DMSO. It was added to cells in PB up to a final concentration of 0.05 mM; after 10 min of incubation, the cells were washed, resuspended in 100 µM of PB and observed by epifluorescence microscopy (Axio Imager.Z1, Carl Zeiss, Oberkochen, Germany) using Zen 2.1 software and a DAPI-specific filter EX BP 365/12, BS FT 395, EM LP 397.

### 5.9. Visualization of Autophagosomes Using MCD and of Lipid Droplets Using Nile Red

Staining of neutral lipids was performed with a lipophilic dye Nile Red (Sigma-Aldrich, St.-Louis, MO, USA) according to [44] with some modifications. Cells were fixed in 4% PFA in 0.1 M PB at pH 7.4 for 15 min and washed with PB by centrifugation at 600× *g* for 5 min. Supernatant was discarded and EDTA was added up to final concentration of 3.75 mg/mL to improve permeability of cells to Nile Red. After 1 min of incubation with EDTA cells were stained by 4 μM Nile Red (prepared from 1 mM stock solution in methanol) for 2 min in darkness at 35 °C and washed twice with OHM growth medium (600× *g*, 5 min). Cells were imaged using confocal laser scanning microscope LSM 780 and Zen 2.1 software (Carl Zeiss, Oberkochen, Germany). Nile Red fluorescence was excited with a 561 nm laser line at 10% of the maximal intensity, and the signal was detected in the range of 570–595 nm.

### 5.10. TEM Studies

Cells taken directly from suspension, as well as cells fixed in a mixture of 2.5% (*v*/*v*) glutaraldehyde and 2% (*w*/*v*) paraformaldehyde in 0.064 M potassium buffer (pH 7.4), were analyzed. Suspension of living cells was left for one h for sedimentation. Cell suspensions were frozen in aluminum plates type A (Ø 6 mm) submerged in 1-hexadecene (Sigma-Aldrich, St.-Louis, MO, USA) using Leica HPM100 (Leica Microsystems GmbH, Wetzlar, Germany). Freeze substitution was proceeded in anhydrous acetone with 1% OsO_4_ (EMS-Chemie Holding AG, Herrliberg, Switzerland), 0.2% uranyl acetate (Structure Probe, Inc., West Chester, PA, USA) for 64 h in AFS2 (Leica). Samples were 3 times washed with acetone and embedded in EmBed 812 epoxy resin (EMS-Chemie Holding AG, Herrliberg, Switzerland). Ultrathin sections were made using ultratome EM UC7 (Leica Microsystems GmbH, Wetzlar, Germany) and stained on grids with 2% uranyl acetate (in water) and Reynold’s lead citrate. Sections were photographed under transmission electron microscope JEM-1400 (Jeol, Tokyo, Japan) equipped with side-mounted camera Veleta (Olympus Corporation, Tokyo, Japan) at 80 kV, or Jeol JEM 2100 equipped with bottom camera Gatan UltraScan 4000 (Gatan Inc., Pleasanton, CA, USA) at 160 kV.

### 5.11. Determination of Astaxanthin Contents

The method described by Li and collaborators [45] was used with some modifications. On day 5 of experiments, 1 mL of culture was taken from each flask. The cells were collected by centrifugation at 24 °C and 600× *g* for 5 min and extracted with 300 µL of DMSO at 70 °C for 5 min with gentle shaking. The cells were centrifuged again and the supernatants were collected. Concentration of astaxanthin in the extracts was determined with spectrophotometer UV-2600 (Shimadzu Corporation, Kyoto, Japan) at 530 nm, using the absorption coefficient of 155,600 M^−1^ cm^−1^ and the equation given by Li and collaborators [45].

### 5.12. Statistics

Five independent experiments were performed, and means of five experiments ± SD are shown if not indicated otherwise. The levels of K^+^ and Na^+^ levels in *H. lacustris* cells were analyzed in three experiments with normal OHM and in one experiment where OMH was additionally supplemented with CaCl_2_, respectively. If not indicated otherwise, statistical significance of the differences was evaluated by means of one-way ANOVA and post hoc Tukey test. Statistical analysis and data visualization were performed using RStudio 3.6.3 [46].

## Figures and Tables

**Table 1:** Photosynthetic performance (JIP-tests parameters) in cells of *Haematococcus lacustris* on days 1 and 3 of salinity treatment. F_O_ (relative units, r. u.)–minimal fluorescence in the dark-adapted state at open PSII reaction centres; F_M_ (r. u.)–maximal fluorescence in the dark-adapted state at closed PSII reaction centres; φ_O–_maximal quantum yield of PSII in the dark-adapted state, similar to F_V_/F_M_; T_FM_ (ms)–time to reach maximal fluorescence F_M_ which characterizes the rate of energy transfer from PSII reaction centres to the PQ pool; S_M_ (ms)–normalized area above the OJIP curve after double normalization (F_O_ = 0, F_M_ = 1), which is a proxy of the number of electron carriers per electron transport chain; V_J_ = (F_J_ − F_O_)/(F_M_–F_O_)–relative variable fluorescense at step J (2 ms) which reflects the proportion of closed PSII reaction centres; V_I_ = (F_I_ − F_O_)/(F_M_ − F_O_)–relative variable fluorescense at step I (30 ms) which reflects the intermediate stationary level of reduction of PQ pool and the electron acceptors beyond PQ; ψ_O_ = (1 − V_J_)–probability of electron transport beyond Q_A_ reflecting the level of oxidation of electron transport chain. Mean values ± SD are shown. Different letters indicate significant differences according to one-way ANOVA and post hoc Tukey test at *p* < 0.05 or less. Bold letters indicate significant differences relative to control cells.

	Control	0.2% NaCl	0.2% NaCl + AZD	0.8% NaCl	0.8% NaCl + AZD
	1 Day				
F_O_	0.04 ± 0.01 ^a^	0.05 ± 0.01 ^ac^	**0.06 ± 0.01 ^bc^**	**0.06 ± 0.003 ^bc^**	**0.08 ± 0.001 ^b^**
F_M_	0.21 ± 0.05 ^a^	0.20 ± 0.02 ^ab^	0.21 ± 0.02 ^a^	**0.14 ± 0.004 ^b^**	0.16 ± 0.004 ^ab^
φ_O_	0.81 ± 0.01 ^a^	0.76 ± 0.03 ^ab^	**0.71 ± 0.01 ^b^**	**0.54 ± 0.03 ^c^**	**0.51 ± 0.02 ^c^**
T_FM_	114.92 ± 4.90	117.46 ± 2.87	119.44 ± 0.68	111.16 ± 0.47	106.9 ± 13.12
S_M_	20.17 ± 3.11 ^a^	18.55 ± 2.08 ^a^	19.83 ± 0.79 ^a^	**7.22 ± 0.64 ^b^**	**8.47 ± 0.06 ^b^**
V_J_	0.33 ± 0.04 ^a^	0.39 ± 0.03 ^a^	0.39 ± 0.04 ^a^	**0.50 ± 0.03 ^b^**	**0.50 ± 0.04 ^b^**
V_I_	0.68 ± 0.05	0.70 ± 0.06	0.68 ± 0.03	0.62 ± 0.03	0.66 ± 0.07
ψ_O_	0.67 ± 0.04 ^a^	0.61 ± 0.03 ^a^	0.61 ± 0.04 ^a^	**0.50 ± 0.03 ^b^**	**0.50 ± 0.04 ^b^**
	3 Day				
F_O_	0.06 ± 0.01 ^ab^	0.05 ± 0.007 ^ab^	0.07 ± 0.001 ^a^	**0.05 ± 0.007 ^b^**	0.07 ± 0.002 ^a^
F_M_	0.18 ± 0.02 ^a^	0.19 ± 0.02 ^a^	0.17 ± 0.005 ^a^	**0.07 ± 0.009 ^b^**	**0.10 ± 0.002 ^b^**
φ_O_	0.67 ± 0.05 ^ab^	0.72 ± 0.02 ^a^	0.62 ± 0.03 ^b^	**0.32 ± 0.03 ^c^**	**0.30 ± 0.01 ^c^**
T_FM_	216.67 ± 12.95 ^ac^	188.4 ± 2.74 ^ac^	232.54 ± 7.01 ^abc^	281.8 ± 48.74 ^ab^	**289.39 ± 19.20 ^b^**
S_M_	18.50 ± 3.34 ^a^	17.99 ± 0.81 ^a^	15.44 ± 0.96 ^ab^	**12.47 ± 1.44 ^b^**	**12.58 ± 1.16 ^b^**
V_J_	0.42 ± 0.11 ^a^	0.41 ± 0.02 ^a^	0.52 ± 0.02 ^ab^	0.54 ± 0.08 ^ab^	**0.64 ± 0.03 ^b^**
V_I_	0.82 ± 0.02	0.82 ± 0.02	0.84 ± 0.005	0.85 ± 0.09	0.88 ± 0.05
ψ_O_	0.58 ± 0.11 ^a^	0.59 ± 0.02 ^a^	0.48 ± 0.02 ^ab^	0.46 ± 0.08 ^ab^	**0.36 ± 0.03 ^b^**

## Data Availability

Data is contained within the article.
